# Human Research Protection Training Requirements: Alternatives for Community Researchers at CTSA‐Funded Research Institutions

**DOI:** 10.1002/eahr.60030

**Published:** 2025-11-12

**Authors:** Emily E. Anderson, Alison Santiago

**Affiliations:** ^1^ Professor at the Neiswanger Institute for Bioethics at Loyola University Chicago; ^2^ Senior regulatory specialist at the Center for Clinical and Translational Science at the University of Illinois Chicago

**Keywords:** research ethics, human research protections, community engagement, community‐engaged research, community researchers, human research protections training, clinical and translational science

## Abstract

Enhanced protection for research participants is often stated as a goal and potential benefit of engaging community partners in research. However, many community‐engaged research teams report challenges in completing human research protection (HRP) training. Some human research protection programs (HRPPs) require community researchers to complete the same training that university faculty and staff members are required to do, while others allow alternatives that are more accessible and appropriately suited to community researchers’ background and roles. To learn more about general HRPP requirements for training and alternatives for community researchers, we surveyed 163 research institutions funded by the National Institutes of Health's Clinical and Translational Science Award program. Out of 55 respondents, 34 HRPPs (61.8%) allow community researchers to complete different HRP training from that required for faculty/staff. Reasons for allowing or denying alternative training vary, and some institutions that currently do not allow alternatives report that they are reconsidering their policy. The fact that some institutions do not allow alternatives and are not considering changing their policies, even when requested by the principal investigators of studies, may pose significant barriers to community‐engaged research. Effort is needed to increase awareness about alternatives as well as their acceptability in meeting training requirements of federal funding agencies.

One often‐stated goal and potential benefit of engaging community partners in research is that community engagement can enhance protections for individual research participants and for communities by providing transparency, improving identification and assessment of risks, and strengthening the informed consent process.^1^ It is then perhaps ironic that many community‐engaged research (CEnR) studies report facing challenges in navigating the institutional review board (IRB) submission and review process.^2^ Moreover, completing human research protections (HRP) training has been widely reported as a particular challenge for CEnR teams.^3^

In establishing a federalwide assurance with the Office for Human Research Protections (OHRP), an institution that conducts research with humans agrees that their human research protection program (HRPP) will “educ[ate] the members of its research community in order to establish and maintain a culture of compliance with Federal regulations and institutional policies relevant to the protection of human subjects.”^4^ No specific HRP training programs are required or endorsed by federal funders of human subjects research or by federal agencies that regulate human subjects research. Research institutions have a great deal of discretion to structure their HRP training programs, and institutional requirements may vary significantly depending on the kinds of human subjects research performed at the institution and the sources of funding for such research.^5^ At a minimum, topics covered generally include the key ethical principles of beneficence, justice, and respect for persons as detailed in the *Belmont Report* ^6^ and U.S. regulatory requirements governing research to fulfill these principles.^7^ HRP training is thus expected to take several hours to complete.

As a general rule, anyone who interacts with research participants or their data is required to complete HRP training, whether principal investigators (PIs) or other research personnel. Some research institutions require completion of online training by commercial organizations (such as the HRP training offered by the Collaborative Institutional Training Initiative [CITI Program]),^8^ while others develop and deliver their own HRP training programs. Often, in CEnR, community members—individuals who may not have previous experience conducting research but have ties to the community where the research is being conducted—are part of the research team with roles in recruiting participants, obtaining informed consent, delivering interventions, collecting and/or analyzing data, and disseminating research findings, and thus require HRP training.

Prior research has identified barriers to HRP training for community researchers.^9^ Standard training programs required by some HRPPs can be poorly suited given community researchers’ limited familiarity with research terminology; these programs are often overly text heavy, which is not compatible with adult learning practices and are geared toward individuals with previous graduate level training in research methods.^10^

More critically, standard HRP training is focused on decisions that arise in research design, such as determining participant inclusion/exclusion criteria or what information should be included in the informed consent form, rather than on ethical and other dilemmas that may occur in day‐to‐day interactions with research participants. Standard training often does not address unique considerations that arise in CEnR, such as recruiting individuals with whom researchers or others on the research team have a prior relationship or maintaining confidentiality in public research settings.^11^ The roles of community researchers often involve direct participant contact through such tasks as recruiting research participants, obtaining informed consent, and collecting data.^12^ Moreover, community researchers may face unique ethical challenges when conducting research in their own communities.^13^ Importantly, community researchers sometimes contribute to research design and serve in investigator roles, and other research personnel could benefit from training more focused on the “frontline” work of research; however, these issues are particularly salient for community researchers working in their own communities.

Community engagement in health‐related research has been increasing, and some research funders (e.g., the Patient‐Centered Outcomes Research Institute) require researchers to incorporate some form of community engagement in their research. The trend toward more community engagement in research—especially in biomedical research—will likely continue, making more urgent the need for appropriate HRP training for community researchers. Community researchers should receive HRP training that meets their needs, takes their unique positions in research and in their communities into account, and addresses the challenges that they are likely to encounter during study implementation. Yet, some research institutions require community researchers to complete the same training that university faculty and staff members are required to do,^14^ even though there are training programs that are more accessible and appropriately suited to community researchers’ background and roles. The number and percentage of institutions that allow community researchers to complete an alternative training program, and the reasons for doing so, are unknown. To learn more about general HRPP requirements for training for all researchers, as well as alternatives for community researchers, we surveyed HRPP directors at research institutions funded by the National Institutes of Health's (NIH) Clinical and Translational Science Award (CTSA) program. HRPP directors were selected as the individuals with the greatest influence on the selection of approved HRP training programs at an institution. Institutions funded through the NIH CTSA program were selected because this program provides research infrastructure and training support to a national network of biomedical institutions with the goal of speeding the translation of research discoveries into improved care for patients, and CTSA‐funded institutions have a mandate to engage local communities in research. Thus, the IRBs at CTSA‐funded institutions should have experience reviewing studies that engage community researchers in roles that require HRP training. Additionally, because these are large academic medical centers, they generally have large and well‐resourced HRPPs, and often their behavior influences what becomes viewed as best practice.^15^

## STUDY METHODS

We sent a brief survey via REDCap to publicly available email addresses of HRPP directors (or their delegates) at 163 institutions affiliated with CTSA hubs, including 64 primary CTSA awardee institutions and 99 “hub affiliates.” Contacts were sent three emails before being considered nonresponsive.

The survey asked about general HRP training requirements for faculty, staff, and students involved in research; whether there are specific policies or requirements for community researchers; whether alternative training requirements for community researchers have been considered; and, if applicable, which alternative HRP training programs for community researchers are allowed and/or endorsed. If an institution responded that they did not allow alternative training for community researchers, we also asked whether alternatives had been considered, whether PIs at the institution had ever requested that alternative training be allowed, and reasons for not allowing alternative training.

Data were downloaded from REDCap into Excel and analyzed using simple statistics such as frequencies, percentages, and cross‐tabulations. Fill‐in responses were recoded to calculate frequencies. This study was determined to meet the criteria for exemption by the IRB/Office for the Protection of Research Subjects at the University of Illinois Chicago (#2024‐0234).

## STUDY FINDINGS

The survey response rate was 33.7% (55/163), typical of similar surveys of HRPP/IRBs and their personnel.^16^ Respondents represented 33 CTSA primary awardee institutions and 22 affiliate institutions; overall, 41 different “hubs” were represented (out of 64 total hubs funded at the time of the survey).

Of the 55 institutions that responded, 52 (94.5%) require faculty, staff, and students to complete training developed by the CITI Program. Those that do not require one of the CITI Program's courses use the OHRP's Human Research Protection Foundational Training program or a training program developed at their institution. Sixteen (30.8%) of the 52 that use the CITI Program's courses also offer other alternatives to fulfill the initial HRP training requirement.

Respondents reported how often their institution requires individuals engaged in human research to complete continuing training. The most common response was every three years (42, 76.4%), with a range of never/no requirement to every five years.

Thirty‐four HRPPs (61.8%) allow community researchers to complete different HRP training from that

**Standard training often does not address unique considerations that arise in CEnR, such as recruiting individuals with whom researchers or others on the research team have a prior relationship or maintaining confidentiality in public research settings.**

required for faculty/staff. Of those 21 institutions that do not currently allow alternative HRP training for community researchers, 12 are considering alternatives; at 8 (38.1%) institutions, allowing an alternative has been requested by a PI (see table 1).

**Table 1:** Do CTSA‐Funded Academic Research Institutes Allow Alternative Human Research Protections Training for Community Researchers? (N = 55)

Status	N (%)
Allow alternatives	34 (61.8%)
Do not allow alternatives	21 (38.2%)
Considering allowing alternatives	12 (57.1%)
Not considering allowing alternatives	7 (33.3%)
Not sure if alternatives have been considered	2 (9.5%)

The most frequently used alternative is CIRTification Online, a free web‐based HRP training program for community researchers developed with NIH CTSA funding at the University of Illinois Chicago's Center for Clinical and Translational Science.^17^ CIRTification is used by half (17) of the 34 institutions that allow alternatives. See table 2 for information about alternative training programs available to community researchers.

**Table 2:** Alternative HRP Training Programs for Community Researchers Used by CTSA‐Funded Academic Research Institutions

Training program	N = 34* (%)
CIRTification Online	17 (50%)
Research Ethics for All	4 (11.8%)
Something developed at their institution	4 (11.8%)
PI is allowed to propose or to develop/deliver, assessed on case‐by‐case basis	7 (20.6%)
CITI‐specific modules only	6 (17.6%)
Custom training	5 (14.7%)
OHRP Human Research Protection Foundational Training	1 (2.9%)

* Respondents were able to choose more than one alternative, so percentages add up to more than 100%.

Respondents from institutions that do not allow alternative HRP training for community researchers cited several reasons for this restriction (see table 3), the most common being that “Anyone working on a human research study must complete the same required training” (12/21, 57.1%). Seven respondents noted they are “not aware of any alternative training programs in human research protections for community research partners.”

**Table 3:** Reasons for Not Allowing Alternative HRP Training for CRs

Reason	#(%) (n = 21*)
Our HRPP/IRB does not review very much community‐engaged research.	3 (14.3%)
We are not aware of any alternative training programs in human research protections for community research partners.	7 (33.3%)
Anyone working on a human research study must complete the same required training.	12 (57.1%)
Per institutional policy, we do not allow alternatives.	2 (9.5%)
We have reviewed alternative training program(s) for community research partners but the quality of the content is not sufficient.	2 (9.5%)
We have reviewed alternative training program(s) for community research partners but decided against using it/them because they are too difficult to track.	1 (4.8%)
Other	6 (28.6%)

* Respondents were able to choose more than one reason, so percentages add up to more than 100%.

## DISCUSSION

Our study is limited by our low response rate, but this rate is similar to the response rate for other surveys of IRBs/HRPPs, a somewhat difficult population to engage in research.^18^ Another limitation of this study is that CTSA‐funded institutions are not representative of all institutions where CEnR is conducted, which limits the generalizability of our results to all universities and other research institutions. However, the list of CTSA awardees does include most large academic medical centers that have relatively well‐resourced HRPPs, and CTSAs have a mandate to engage community partners, and thus likely provide reliable information regarding trends in HRP training for community researchers.

Most of the institutions we surveyed require faculty and staff researchers to complete the CITI Program's HRP training. Requirements for continuing education varied, but almost all required continuing education (“refresher” training) at some point. Additionally, we found that many but not all HRPPs at CTSA‐affiliated institutions allow community researchers to complete HRP training that is different from that required for faculty and staff and developed specifically for CEnR. Reasons for allowing or denying alternatives vary across institutions, and some institutions that currently do not allow alternatives report being in the process of reconsidering their policy. This is encouraging and could reflect a shift in HRPP knowledge and attitudes toward facilitation of CEnR. However, the fact that some institutions do not allow alternatives and are not considering changing their policies, even when requested by PIs, is troubling as this may pose significant barriers to CEnR. Policies and procedures reflect an institution's commitments and values, and ideally these will encourage and support CEnR.^19^

One curious finding is the number of respondents (12) that stated that their institution does not allow alternative training for community researchers because of a perception that “everyone working on a research study must complete the same required training.” Unfortunately, it is unclear if this reflects a misguided belief that there is a legal/regulatory requirement for a particular training program or consistency in training, or just a factual statement about what happens at their institution, perhaps for administrative ease. To be sure, tracking different requirements for different research personnel will add burden to HRPP operations, which are already under‐resourced and stretched thin.^20^

We were also surprised at the number of respondents (7) who indicated that they were not aware of any alternative HRP training programs suitable for community researchers. While it may be that other individuals at their institution know of alternatives, this suggests that in tandem with messaging about the regulatory acceptability as well as the benefits of alternative training for community researchers, there is a need to promote awareness of available resources. A range of alternative HRP training programs has been developed at various institutions to meet the unique needs of community researchers working on different projects and representing different populations.^21^ Resources to support implementation of these options as well as evaluate and continuously update and improve them are critically needed.

Quality HRP training promotes the ethical conduct of research. It is not just a hoop that those engaged in research must jump through; it is meant to provide knowledge and skills to researchers conducting studies with human participants. To adhere to the ethical principles of CEnR, researchers must “identify and mobilize community assets and strengths through developing the community's capacity and resources to make decisions and take actions.”^22^ This includes ensuring community researchers have access to meaningful and engaging professional development opportunities, including HRP training. Training that is a poor fit for community researchers’ needs given their background and day‐to‐day research responsibilities may result in teams not having the tools and knowledge they need to do their jobs well. Training that is a poor fit may also diminish the perceived value of ethics, human research protections, and the role of the IRB in oversight of human subjects research. HRP training influences how research personnel think about research integrity and participant safety and well‐being in relation to other important “goods”—such as enrolling participants. It is therefore critical that relevant, quality HRP training for community researchers be accessible and supported by partner academic institutions.

## ACKNOWLEDGMENT

This research was supported by the National Center for Advancing Translational Sciences, NIH, through grant UL1TR002003. The content is solely the responsibility of the authors and does not necessarily represent the official views of the NIH.

## DISCLOSURE

Emily E. Anderson is the author and creator of CIRTification Online.
